# Catatonia in Older Adult Individuals with Intellectual Disabilities

**DOI:** 10.1155/2015/120617

**Published:** 2015-10-01

**Authors:** Megan White, Edward Maxwell, Warren E. Milteer, Jose de Leon

**Affiliations:** ^1^Department of Psychiatry, College of Medicine, University of Kentucky, Lexington, KY 40509, USA; ^2^Hazelwood Center ICF/IID, Louisville, KY 40215, USA; ^3^University of Kentucky Mental Health Research Center, Eastern State Hospital, Lexington, KY 40511, USA; ^4^Psychiatry and Neurosciences Research Group (CTS-549), Institute of Neurosciences, University of Granada, 18971 Granada, Spain; ^5^Biomedical Research Centre in Mental Health Net (CIBERSAM), Santiago Apóstol Hospital, University of the Basque Country, 01004 Vitoria, Spain

## Abstract

Catatonia has been described in children with intellectual disabilities (IDs). These are the first three published cases of catatonia in adults older than 50 years of age with IDs. They were followed using the KANNER scale and, in one case, creatinine phosphokinase (CPK) monitoring. Case 1 is a 67-year-old Caucasian who probably had been having intermittent episodes of undiagnosed catatonia withdrawal for many years. His episodes of agitation and withdrawal behavior responded to lorazepam up to 8 mg/day. Case 2 is a 63-year-old Caucasian male who had probably had undiagnosed catatonic episodes since age 25. An agitation episode that rated 88 on Part 2 of the KANNER scale ended within minutes after he received 1 mg of intramuscular lorazepam. He had no symptom relapses for 4 years after getting stable oral lorazepam doses (3–8.5 mg/day). Case 3 is a 55-year-old African-American male with severe ID and bradycardia (with a pacemaker). He had been “institutionalized” since age 22 and his undiagnosed catatonic episodes appeared to have been intermittently present for at least the last ten years. As he became tolerant and experienced symptom relapse, oral lorazepam was slowly increased (1.5–18 mg/day). Electroconvulsive therapy was ruled out due to his pacemaker.

## 1. Introduction

Although some psychiatrists think that catatonia has disappeared, it is becoming apparent that many psychiatrists underdiagnose catatonia both inside [1] and outside the United States [2, 3]. One of the contributing factors to this underdiagnosis may be the ever-changing conceptualization of catatonia (Table 1). Initially Kahlbaum described it in 1874 [4, 5] as a distinct entity, but Kraepelin mainly considered catatonia as a subtype of schizophrenia (dementia praecox) [6]. After Kraepelin, many authors disagreed with this opinion [7–10] but the Diagnostic and Statistical Manual of Mental Disorders 3rd edition (DSM-III) [11] and the revised third edition (DSM-III-R) [12] followed Kraepelin's hypothesis and included catatonia as a type of schizophrenia. The influence of new publications [13–18] led the authors of the DSM-IV [14] and its text revision, DSM-IV-TR [17], to propose that catatonia consists of three entities: a type of schizophrenia, a specifier for mood episodes, or a disorder secondary to medical conditions. In the US, Taylor and Fink were the main defenders of catatonia as a separate diagnostic category [1, 13, 16, 18]. The DSM-5 eventually followed their recommendations and categorized catatonia according to three subtypes: (1) catatonia associated with another mental disorder (catatonia specifier), (2) catatonic disorder due to another medical condition, and (3) unspecified catatonia [19]. Not all experts agree with the DSM-5 decision [20].

The development of catatonia scales has contributed to an improved definition of catatonic symptoms and how to assess them.  Table 2 describes the published catatonic scales [21–31], with particular emphasis on the KANNER scale [31], which was used to assess symptoms in the three patients described in this report. In our experience, the KANNER scale is a good choice for training residents to assess catatonic signs and is helpful for nurses (or other staff) who are quite familiar with the patient to assess some of the core signs and symptoms for long-term monitoring. Although it has not been systematically studied, several articles [32–34] verified our experience that creatinine phosphokinase (CPK) monitoring is an additional objective way to monitor the severity of catatonia.

There is no agreement concerning the physiopathological mechanisms that underlie the catatonic syndromes. Most authors tend to agree that catatonic symptoms reflect frontal lobe dysfunction [1, 18]. Dhossche et al. [35] provided a comprehensive review of the possible contribution of different etiopathogenic mechanisms. Lauterbach et al. [33, 36] have explored the contribution of different neurotransmitters. In that sense, NMDA neurotransmission may be important, given that anti-NMDA-receptor encephalitis frequently manifests catatonic symptoms [37]. Rosebush and Mazurek [38] have proposed that “extreme fear” may be a common contributing factor in all catatonic states.

Due to the severity and relative rarity of the condition, it is not easy to conduct randomized clinical trials (RCTs) in catatonic patients. There are only three RCTs published regarding catatonia [39–41]. The first RCT was in acute catatonia, where McCall et al. [39] randomized ten patients with catatonic mutism to an intravenous (IV) amobarbital solution and ten patients to IV saline. The respective initial response rate was 6/10 and 0/10. Four of the saline nonresponders had a response when given amobarbital. The other two were negative RCTs (one with lorazepam [40] and one with amineptine [41]) in patients with schizophrenia and enduring chronic catatonic symptoms. It is possible that chronic catatonic symptoms in schizophrenia may be different than the typical cases of acute catatonia more frequently encountered by clinicians in the US. There is general agreement in the literature that there are two major types of treatments for acute catatonia [42–44]: sedative agents in large dosages (frequently in parenteral form) and electroconvulsive therapy (ECT). Among sedatives, amobarbital was initially used, but more recently benzodiazepines have been substituted [44]. Similarly, chemically induced seizures were first used in patients who were catatonic, but this was rapidly replaced by ECT, which is now considered the treatment of choice when there is no response to benzodiazepines [42–44]. Sometimes catatonic patients appear to become tolerant to benzodiazepines, but they may recover their benzodiazepine response after ECT [45].

Classical authors [46, 47] described catatonia in children with mental retardation (currently called intellectual disabilities [IDs]) and/or autism. Then in the 2000s [48–55] more and more articles appeared reporting that catatonia can be associated with IDs and autism in patients without an underlying diagnosis of schizophrenia or mood disorders, thus leading to the inclusion in the DSM-5 of neurodevelopmental disorders among the other mental disorders associated with catatonia [19]. Review articles of the limited available information suggest that these catatonic patients with IDs may also respond to benzodiazepines and/or ECT [55]. Clearly, some cases of ID are associated with treatable conditions [56]. It would stand to reason that correct identification and treatment of an underlying neuropsychiatric illness (such as Wilson's disease, anti-NMDA receptor encephalitis, multiple sclerosis, PANDAS, lupus encephalopathy, HIV, and Fahr disease) would also result in the treatment of catatonic symptoms associated with the underlying illness. Unfortunately, the literature provides almost no information regarding treatment for the underlying neuropsychiatric conditions versus “symptomatic treatment” with benzodiazepines and/or ECT [57].

Recognizing catatonia as a unique clinical entity rather than as a modifier for schizophrenia or mood disorders allows for more rapid diagnosis in patients who have an alternative underlying diagnosis (such as autism and/or an ID) and allows clinicians to more accurately diagnose and more rapidly treat such patients [50]. Overall, there is a paucity of modern psychiatric literature describing catatonia in individuals with an autism spectrum disorder or an ID, and there are few published cases of catatonia in adults with IDs, according to two systematic treatment reviews of the literature [53, 55]. Mazzone et al. described eight published cases in adults with no ages provided [53]. DeJong et al. identified only six cases in adults with IDs older than 20 years old and only one case older than 30 years of age (35 years old) [55]. In a recent editorial, Dhossche [54] commented on the lack of studies of older patients defined as >60 years of age.

This paper describes three cases of catatonia in older male adults with IDs who were followed using the KANNER scale [31] after benzodiazepine treatment. Mr. A was 67 years old when diagnosed with catatonia but had been seen for psychiatric symptoms since he was diagnosed with profound mental retardation at age 6 months. After the catatonia diagnosis and the lorazepam response, the mother reported that the patient had been having intermittent episodes of catatonia withdrawal for many years. Mr. B was 63 years old when diagnosed with catatonia, but a chart review indicated that episodes of catatonia were present since at least age 25. Mr. C was 55 years old when diagnosed with catatonia. He had been “institutionalized” since he was 22 years of age and it was unclear when the catatonic episodes started, but they appear to have been intermittently present over the last ten years.

## 2. Case Presentations

### 2.1. Case  1

#### 2.1.1. Prior History

Mr. A is a 67-year-old Caucasian male. He had a history of severe jaundice at birth due to Rh factor incompatibility and was diagnosed with kernicterus. Currently, kernicterus is a controversial diagnosis, and some prefer using the words “bilirubin-induced neurologic dysfunction” [58] to describe this syndrome. He was diagnosed with “mental retardation” by the age of six months, did not ever develop useful language, and was diagnosed with profound mental retardation. He was admitted to an institution for children with IDs. He badly burned himself in a cooking accident at the age of 34. Mr. A also had a history of epilepsy but had been seizure-free since age 61. He had many psychiatric admissions as an adult. He had been continuously admitted to the same hospital for the last 17 years, prior to being evaluated.

The patient had multiple past psychiatric diagnoses, but never catatonia. He had been treated with many psychiatric medications from all the psychiatric classes (antidepressants, anticonvulsants, antipsychotics, and stimulants) except for long-term treatment with high doses of benzodiazepines.

Upon review of his chart, it was notable that Mr. A was prescribed lorazepam 2 mg intramuscularly every 4 hours as needed for “agitation.” His episodes of agitation at that time were described as “flailing, kicking, and meal refusals.” After administration of lorazepam, he would calm markedly and would eat his meals. Interestingly, he did not appear sedated after receiving lorazepam but rather was alert, oriented, and engaged. He would remain calm for about three to five hours after the dose of lorazepam and then another episode of agitation would reoccur, often resulting in the administration of a repeat dose of lorazepam. Two years after admission, his as-needed medication was changed from lorazepam to chlorpromazine, and it appears that this was not overly effective. After further chart review, he was noted to have episodes of “deep depression” that were treated with citalopram. During these episodes he would pull away from favored activities that he seemed to enjoy and would withdraw, sulk, sleep poorly, and stop eating. After the catatonia diagnosis, it was considered possible that these episodes of “depression” might actually be episodes of catatonic withdrawal and that his flurries of intense and challenging behavior may be episodes of catatonic excitement.

The description of the symptoms in the medical records was very poor, so we cannot establish when the catatonic symptoms began in this patient. After the remarkable improvement from high doses of lorazepam, which not only helped with agitation but eliminated periods of catatonic withdrawal, the mother stated that the patient has had intermittent episodes of catatonia withdrawal for many years until a high dose of lorazepam was started at 67 years of age. In summary, the patient had many psychiatric symptoms throughout his life and, according to his mother, continued to have catatonic withdrawal episodes for many years until he was treated with high-dose lorazepam.

#### 2.1.2. Assessment

The senior author was asked to see the patient to assess whether his uncontrollable agitation could be explained by catatonia. At that time, the patient was taking 1800 mg/day of gabapentin for arthritic pain and 20 mg/day of citalopram. The prior psychiatrist had started citalopram at least 4 years prior. The current psychiatrists thought the episodes of withdrawal were not a sign of depression but of catatonia withdrawal.

At the time of assessment, Mr. A was having episodes of agitation that were irregular in frequency and were occurring about two times per week. Approximately half of the time no trigger could be identified for the behaviors; they would last from minutes to a full day. When his episodes were very severe, it was difficult to hold the patient, and he would require up to four adult staff to hold his extremities to prevent injury. He was also described as having abnormal posturing and progressive sequences of arm postures such as holding his elbow at a 90-degree angle. Mr. A exhibited sequences of movements in his arms and legs, such as hitting his shin with his heel repetitively. Such movements would increase with agitation.

On the initial exam performed by the senior author, he was nonverbal and did not follow simple commands. He was reclining in his wheelchair with his back arched. His left arm maintained a folded position behind his head and his chair for five to ten minutes. His right arm was positioned in front of him and placed at a 90-degree angle, and he held it there for several seconds. Most of the time he did not demonstrate stiffness or resistance when his arms were moved; however, occasionally, he showed resistance that was proportional to the efforts to move him, consistent with paratonia. He also had features compatible with a generalized tremor that was consistent with a cerebellar lesion (which was thought to correlate with his diagnosis of kernicterus). He also had severe dyskinetic movements in his face and lower extremities, possibly secondary to previous antipsychotic use or to the basal ganglia damage associated with kernicterus. He had no dystonia, nor chorea or athetoid movements.

It was determined that he met the following eight out of the twelve criteria for catatonia: stupor, waxy flexibility, mutism, negativism, posturing, mannerism, stereotypy, and agitation not influenced by external stimuli. He was diagnosed using the DSM-IV-TR [7] as having Catatonic Disorder due to a General Medical Condition (kernicterus) given that, at that time, the DSM-IV-TR did not allow a diagnosis of catatonia associated with ID. If the DSM-5 had been in use, he would have been diagnosed with catatonia associated with a neurodevelopmental disorder. He was also diagnosed with two noncatatonic abnormal movements: (1) generalized tremor that was consistent with a cerebellar tremor and (2) dyskinesia secondary to basal ganglia damage from kernicterus versus tardive dyskinesia secondary to previous antipsychotic treatment.

#### 2.1.3. Follow-Up

For treatment, he was started on scheduled oral lorazepam 1 mg/day (0.5 mg twice a day), which was increased up to 4.5 mg/day (1.5 mg three times a day) over a period of two months and then eventually increased to 8 mg/day over the subsequent 12 months. He showed marked improvement, particularly in his posturing and stereotypy, on the KANNER scale. Additionally, it is also interesting to note that, as with most other patients with catatonia, he did not have any symptoms of lethargy or respiratory depression after lorazepam administration, even after increasing his daily dose to 8 mg. The treatment with lorazepam helped him to have some of the best years of his late life until age 72, when he developed aspiration pneumonia and then was transferred to a nursing home.

One year after the assessment, when the patient was doing well on lorazepam, a slow taper of citalopram was started and then it was discontinued completely. In summary, citalopram did not prevent episodes of catatonia withdrawal and its discontinuation did not bring them back.

### 2.2. Case  2

#### 2.2.1. Prior History

Mr. B is a 63-year-old Caucasian male with a history of an ID and epilepsy; he has lived in a group home setting since he was 20 years old. A chart review showed that Mr. B had previously been able to independently perform many activities of daily living until the fifth or sixth grade. He was noted to have had gradual neurological deterioration from age 13 until age 17, with impaired speech, loss of the ability to walk independently, and a change in his seizure pattern. His intelligence quotient (IQ) was estimated to be around 30 at that time.

When he was 25 years old, Mr. B was noted to have periods of hostility, agitation, and aggression for which he was prescribed sodium amobarbital intramuscular injection as needed and oral thioridazine 300 mg/day. He was reported to engage in property destruction such as tearing linens, taking light fixtures and air conditioning units off of the wall, throwing filters on the floor, taking all the drawers out of his dresser and emptying the contents on the floor, turning over furniture, and lifting his bed high enough that the mattress would fall off. Some of these episodes were reported to occur while he was withdrawn, quiet, and sullen, with poor eye contact, psychomotor retardation, hypersomnia, decreased appetite, low energy level, low interest level, and lack of motivation.

When he was 33 years old, he was found with a toothpick pushed into his genitalia that caused bleeding. At that time, Mr. B was on mesoridazine, orally 75 mg/day, and benztropine, orally 4 mg/day. When he was 38 years old, he was admitted for the first time to a psychiatric hospital for evaluation. At that time, he was found unclothed on the sun deck of the group home in what appeared to be an attempt to jump off the brick railing on the third floor. He also was noted to be increasingly withdrawn, staying mostly in bed and refusing to get up, eat, or take his medications. He would stay under his bed for days, refusing to come out of his room. Mr. B was started on carbamazepine and was discharged back to his group home after a month-long hospitalization. At age 41, he was started on doxepin for “recurrent destructive attempts.” The following year, he was evaluated by a psychological consultant after increasingly unusual and bizarre behavior. His speech was marked by delusional themes concerning “the cross” and being crucified. He was also noted to have periods of “posturing and waxy flexibility.” He was described as noncommunicative and rigid, holding his arms up in the air for long periods of time. He also had periods of staring off into space or appearing distracted and not reality-based. Mr. B would also refuse to talk, eat, take his medications, or participate in daily hygiene and grooming. At that time his diagnosis was changed to schizoaffective disorder. Over the next ten years he was tried on multiple different medications including haloperidol, risperidone, venlafaxine, and paroxetine for psychosis and mood lability but continued to demonstrate episodes of withdrawal, abnormal posturing that the staff would call “statue man,” and social isolation, followed by periods of intense agitation with intermittent self-injurious behaviors and aggression towards staff.

Seven months prior to evaluation, Mr. B had an episode of agitation that was rated by the staff using the KANNER scale [31] and was given a score of 88 for Part 2. At that time, he was taking haloperidol, aripiprazole, lamotrigine, and valproic acid. The episode of agitation lasted about ten hours and ended within minutes after he received an intramuscular injection of lorazepam 1 mg (after two previous 0.5 mg doses of oral haloperidol that did not appear to work). During this episode, he was noted to be lying in the middle of the living area naked, trying to bite and kick staff and scratching his shoulder, and he had defecated and smeared feces in his bed, was constantly moving himself off of the safety mats, and was pounding his feet in a rhythmic motion on the floor and was also refusing to eat. Additionally, Mr. B was noted to have a “general blank stare on his face” with pursed lips and squinted eyes and tense facial muscles. He required four staff members to provide him with adequate hydration, nutrition, and medications.

Over the next seven months, Mr. B had at least five milder episodes of similar behavior, all of which showed improvement in behavior after intramuscular injections of lorazepam. Seven days prior to evaluation, he was started on oral lorazepam 3 mg/day (1 mg three times daily). This resulted in a six-week period free from such episodes until he had an episode where he was found to be lying on the floor, mute, and uncooperative. His repeat KANNER score was rated at 46 for Part 2.

#### 2.2.2. Assessment

A psychiatric consultation with the senior author was requested regarding the frequent episodes of agitation that were not controlled with his prior medication regimen. The treating psychiatrist could not wait one week for the evaluation and had started lorazepam. He requested the consult to have his diagnosis of catatonia verified and the chronic treatment with lorazepam approved. After seven days on 3 mg/day of lorazepam, the patient no longer had any catatonic signs. It was determined that during the prior seven months he had met the following eight out of the twelve criteria for catatonia: stupor, mutism, negativism, posturing, mannerism, stereotypy, agitation not influenced by external stimuli, and grimacing. If the DSM-5 had been available at that time, he would have met criteria for catatonia associated with a neurodevelopmental disorder. The patient had no history of depressive or manic episodes and no history of specific schizophrenia symptoms such as bizarre delusions or the typical auditory hallucinations. Additionally, similar to the patient in the previous case, this patient also was noted to have a generalized tremor in his trunk, head, and arms and was diagnosed with a cerebellar tremor.

#### 2.2.3. Follow-Up

Mr. B had no relapse in catatonic symptoms for four years after getting stable doses of lorazepam up to 8.5 mg/day.

### 2.3. Case  3

#### 2.3.1. Prior History

Mr. C was a 55-year-old African-American male born in a neighboring state. He and his brothers were raised by their uncle and aunt. According to the records, his father and mother did have limited education and possibly were mentally retarded but, according to the family report, his parents never had any psychiatric admissions. Mr. C. had two brothers: one possibly had mental retardation and the other was definitively diagnosed with mental retardation and had multiple admissions to our state psychiatric hospitals. According to the records, the patient was malnourished when he was a baby. He did not learn to talk until age 7 when he was diagnosed with mental retardation. His first intelligence quotient (IQ) was 41 at age 8. At age 22, he had his first psychiatric admission due to aggressive behavior and carried the diagnosis of severe mental retardation. Since that time, he had been “institutionalized” and spent most of his time in hospitals for adults with ID with occasional admissions to psychiatric hospitals. According to the records available, he had taken different antipsychotics throughout his life since he was 15 years of age, but there was no clear description of delusions and hallucinations. The antipsychotic appears to have been prescribed to control his behavior, including inappropriate sexual behavior. He had also been tried for several months on lithium and carbamazepine, which appear to have been added to augment his antipsychotic medications in an effort to control his aggressive behavior. At 36 years of age, he was taken to an emergency room for syncope, and an abnormal value of CPK of 415 was described, but the cause could not be identified. There was no description of the behavior at that time to verify or rule out catatonia, and the abnormal CPK was not followed up. Over many years, episodes of unprovoked agitation, sometimes with violence toward others, were described; however, the description was very poor, and it was impossible to retrospectively determine if they were associated with catatonia or not. At the time of the assessment, he had resided at a hospital for adults with IDs for ten years, and his diagnoses were psychotic disorder not otherwise specified, bradycardia (with a pacemaker), and severe mental retardation. The patient had never been diagnosed with catatonia, but once some of the behaviors were identified as catatonic, the staff described these behaviors as having been intermittently present for the last ten years.

#### 2.3.2. Assessment

At the time of initial evaluation, he demonstrated catatonic symptoms such as posturing, mutism, stripping, freezing, posing, mild immobility, fixed staring, grimacing, hitting and rubbing and scratching his bottom to the point of injury, and twisting paper [31]. These behaviors seemed to be stereotypic in nature and did not respond to redirection. He had no other abnormal movements. He had no hallucinations or delusions. Moreover, hallucinations and delusions were not present in the year that the senior author followed Mr. C and were not described by the hospital staff who had known him for years.

It was determined that during the assessment and year of follow-up he met the following eight out of the twelve criteria for catatonia: stupor, mutism, negativism, posturing, mannerism, stereotypy, agitation not influenced by external stimuli, and grimacing. Other catatonic symptoms included in the KANNER scale [31] that were present with catatonic worsening were refusal to eat or drink and nudism. If the DSM-5 had been available at that time, he would have met criteria for catatonia associated with a neurodevelopmental disorder. The patient had no other abnormal movements besides those associated with catatonia.

#### 2.3.3. Follow-Up

Mr. C was started on oral lorazepam, 3 mg/day, and his CPK level at that time was 992 (Table 1), but the lorazepam treatment was initially intermittent. He was followed up for one year for treatment of catatonia with lorazepam and required increasingly higher doses over the course of the year, likely secondary to developing tolerance to the medication. His behavior, CPK, and KANNER scores were monitored. During this time, he was also taking olanzapine 20–25 mg/day. Lorazepam became a standard dose of 1.5 mg/day on day 129 and then increased to 3 mg/day on day 137, to 9 mg/day on day 157, to 12 mg/day on day 199, and to 18 mg/day on day 221 (Table 1). Generally, the patient's behavior would improve for a few days to weeks after increasing his daily dose of lorazepam; however, he would quickly become tolerant of the medication and would have a relapse in symptoms. Furthermore, at times, it was difficult to determine whether his aggressive behaviors were behavioral in nature or were secondary to an underlying etiology such as catatonia.

Despite using increasing doses of lorazepam, the catatonic behavior appeared to become partly tolerant to lorazepam. We have prior experience with patients recovering benzodiazepine response after ECT, which has been described by Petridis et al. [45]. Treatment with ECT was seriously considered when catatonic symptoms including ambitendency [27] and a specific stereotype (see footnotes 1 and 2, Table 3) increased at the same time that there were increases in aggressive behavior and CPK rose to the 1400s. Due to the presence of a pacemaker, ECT was excluded as a treatment option by the only available hospital providing ECT in the area. It should also be noted that, despite these high benzodiazepine doses, he did not become sedated or have any symptoms of respiratory suppression.

## 3. Discussion

The underdiagnosis of catatonia appears to be a major problem [1–3]. In the last 15 years, it has become clear that underdiagnosis of catatonia in children with IDs is also a problem [48–55]. In a recent editorial, Dhossche [54] commented on the lack of studies of older patients, defined as >60 years of age. In the three adults with IDs presented in this case series, the diagnosis of catatonia had been missed for many years. Mr. A, who was 67 years old, probably had intermittent episodes of catatonia withdrawal for many years. Mr. B, who was 63 years old, had definitively had episodes of catatonia since age 25. Mr. C, who was 55 years old, had catatonic episodes intermittently present for at least ten years. He had a high CPK value 19 years earlier, that is, at the age of 36 years, that may have been explained by catatonia.

The above cases demonstrate the importance of elucidating whether symptoms of “agitation” in adult patients with IDs are behavioral in nature or catatonia secondary to an underlying neurodevelopmental disability. In patients with IDs and long history of multiple treatments, such as these patients, placebo responses are unlikely; therefore, it is doubtful that the lorazepam response could be explained by a placebo response. As illustrated in Cases  1 and 2, once the correct diagnosis is identified and appropriate treatment is initiated, the improvement can be dramatic and long-lasting. It should also be noted that all of the above patients were given increasingly higher doses of lorazepam. Despite these seemingly high doses (up to 18 mg/day in Case  3), none of the patients displayed symptoms of sedation or respiratory distress and typically were more alert and interactive after the administration of the medication. Case  3 is an example of the fact that catatonic patients can become tolerant to high doses of benzodiazepines. Some of these tolerant patients may recover a response to benzodiazepine after ECT [45].

The patient discussed in Case  3 was very challenging, due to the consistent reemergence of catatonic symptoms as he developed tolerance to lorazepam. Given his complex behavioral symptoms, CPK was used as an indicator that his symptoms were not volitional but were likely secondary to an underlying biological dysfunction (likely catatonia). Although it was not an exact linear correlation, his elevated CPK level corresponded to worsening symptoms of catatonia. Furthermore, his lowest CPK levels were associated with his lowest KANNER scores, when the patient showed good response to 9 mg/day of lorazepam (Table 3).

It is possible that the KANNER scale does not fully reflect the overall severity of the patient's current state of health. The scale is not weighted for symptoms that could be life-threatening (i.e., not eating or drinking, etc.); therefore, the total KANNER score should be interpreted carefully and the patient's overall state of health should be considered when determining treatment. Table 3 shows that the item “mutism,” which was elevated at the beginning of the follow-up, decreased while the patient was responding to lorazepam doses of 9–12 mg/day but later worsened, which led to an increased dosage of up to 18 mg/day. Future studies need to consider the possibility that CPK could be used as a more objective measure of severity of illness and could be used in conjunction with the KANNER scale. This would be especially helpful in patients with IDs or other neurodevelopmental disabilities who may have baseline behavioral problems that make assessing catatonia difficult. Lastly, it is possible that CPK reflects some catatonic symptoms more than others. Our experience is that the highest CPK elevations in catatonic patients occur with severe exacerbations of hyperactive symptoms of catatonia (i.e., excitement and combativeness) or of immobility with severe stiffness. Future studies need to better establish the relationship of CPK and catatonic symptoms.

## 4. Conclusion

To date, there is no literature discussing older adult patients with IDs and catatonia. This is likely secondary to the underidentification of catatonia as well as the complexity of treating patients with IDs. With the change in the DSM-5 diagnostic criteria, hopefully awareness of catatonia as an entity separate from psychosis will increase awareness of catatonia in adults with IDs. Better tools are needed to help monitor and treat these patients.

## Figures and Tables

**Table 1:** Timeline of important events in the history of the diagnosis of catatonia.

Year	Event	Reference
1874	Kahlbaum described catatonia as a distinct entity.	[4, 5]

1919	Kraepelin described catatonia as a subtype of schizophrenia (dementia praecox), although he also stated it could be found in mood disorders (manic-depressive illness).	[6]

1974	Gjessing published an article in English summarizing his long experience with periodic catatonia, which sometimes is a familial illness.	[7]

1976	Gelenberg published an influential review which suggested that catatonia is a syndrome instead of being a form of schizophrenia.	[8]

1976	Abram and Taylor published their influential prospective study suggesting that the most frequent cause of catatonia in their US hospital was mood disorders.	[9]

1979	Leonhard's textbook fifth edition was translated into English. In his view, catatonic symptoms can be found in (1) motility psychoses (under cycloid psychoses); (2) familial periodic catatonia (under unsystematic schizophrenias); and (3) nongenetic forms of catatonic schizophrenia (under systematic schizophrenias).	[10]

1980	DSM-III included catatonia as a type of schizophrenia.	[11]

1987	DSM-IIIR, as in DSM-III, continues to describe catatonia as a type of schizophrenia.	[12]

1990	Taylor published a comprehensive review on catatonia.	[1]

1991	First article by Fink and Taylor proposing a separate DSM category for catatonia.	[13]

1994	DSM-IV included catatonia as a type of schizophrenia, a specifier for mood episode, and a disorder secondary to a medical condition.	[14]

1997	Peralta et al. studied 567 psychotic patients, identifying 45 catatonic patients, most of whom appear to fit in a separate group, as Leonhard proposed in the motility psychoses.	[15]

2003	Influential article by Taylor and Fink proposing that DSM create a category for catatonia.	[16]

2004	DSM-IV-TR, as did DSM-IV, included catatonia as a type of schizophrenia, a specifier for mood episode, and a disorder secondary to a medical condition.	[17]

2006	Taylor and Fink published an influential book in catatonia.	[18]

2013	DSM-5 described catatonia as separate syndrome. DSM-5 required 3 of 12 catatonic symptoms to make the diagnosis of catatonia. The symptoms are (1) stupor, (2) catalepsy, (3) waxy flexibility, (4) mutism, (5) negativism, (6) posturing, (7) mannerism, (8) stereotypy, (9) agitation not influenced by external stimuli, (10) grimacing, (11) echolalia, and (12) echopraxia.	[19]

**Table 2:** Catatonia scales.

Review	Sienaert et al. [21] completed an excellent review of the published catatonia scales. Kirkhart et al. [22] presented a more critical view of scales and diagnostic criteria.

List of catatonia scales	(i) Rogers Catatonia Scale [23] and its modifications [24–26]
	(ii) Bush-Francis Catatonia Rating Scale [27, 28]
	(iii) Northoff Catatonia Rating Scale [29]
	(iv) Braunig Catatonia Rating Scale [30]
	(v) KANNER scale [31]

Description of KANNER scale	It was developed by Carroll et al. [31] as a “unifying instrument” that could be used across all neuropsychiatric illnesses and consists of three parts.
	(i) Part 1 is used to screen for the presence or absence of catatonia while Parts 2 and 3 are used to monitor symptoms across time.
	(ii) Part 1 is an 11-item screening mnemonic.
	(iii) Part 2: if two or more signs are detected using Part 1 of the scale, Part 2 of the KANNER scale is used, which consists of 18 questions that provide a score between 0 and 144. Nurses or other health providers who are in close contact with the patient usually score Part 2.
	(iv) Part 3 can be used by a psychiatrist to monitor catatonic signs.

**Table 3:** Case 3. Treatment, CPK, and catatonic symptoms.

Day	Olanzapine mg/day	Lorazepam mg/day	CPK IU/L (range 24–204)	KANNER scale		Behaviors
				Total score	Mutism	
1	20	3				
30	20	3	992	92	4	Stereotypies^1^ and ambitendency^2^
31	20	0				
108	20	0	262			
129	20	1.5				
135	20	1.5				
137	20	3		86	2	
157	20	9				Improvement^3^ and ↑ social interactions
191	20	9	692			
198	20	9	776	76	2	↑ aggressive behavior and stereotypies^1^
199	20	12	600			
217	20	12	801			Improvement
221	20	18	442			
239	20	18	682			
242	25	18	408			
246	25	18	416			
248	25	18	408			
253	25	18	721			
262	25	18	441	58	2	
270	25	18	379			
273	25	18	332			
280	25	18	346			
288	25	18	538			
294	25	18	805			Worsening, running away from house
302	25	18	375			
308	25	18	426			
316	25	18	835			
331	25	18	1202	72	6	
345	25	18	1493			↑ aggression, stereotypies^1^, and ambitendency^2^
352	25	18	1460			

^1^Stereotypies: the most characteristic stereotype associated with catatonic symptoms was rubbing and scratching his bottom to the point of irritating the skin.

^2^Ambitendency: the patient appeared motorically stuck in indecisive, hesitant movements [27]. The patient would go back and forth from the shower and get dressed and undressed.

^3^The patient was described as the “best ever” by staff.
